# Supplementary material for: Conditional chemoconnectomics (cCCTomics) as a strategy for efficient and conditional targeting of chemical transmission
Source: eLife. 2024 Apr 30;12:RP91927. doi: 10.7554/eLife.91927 (PMC11060718; doi:10.7554/eLife.91927)
Supplement: Figure 3—source data 3. [file elife-91927-fig3-data3.zip › Figure 3 source data3 part 2.zip!/Figure 3-Source data 3-part2/1062-13-CRISPResso_Report_jt28fhr9.zip!/CRISPResso_on_1062_16/1a.Read_barplot.pdf]

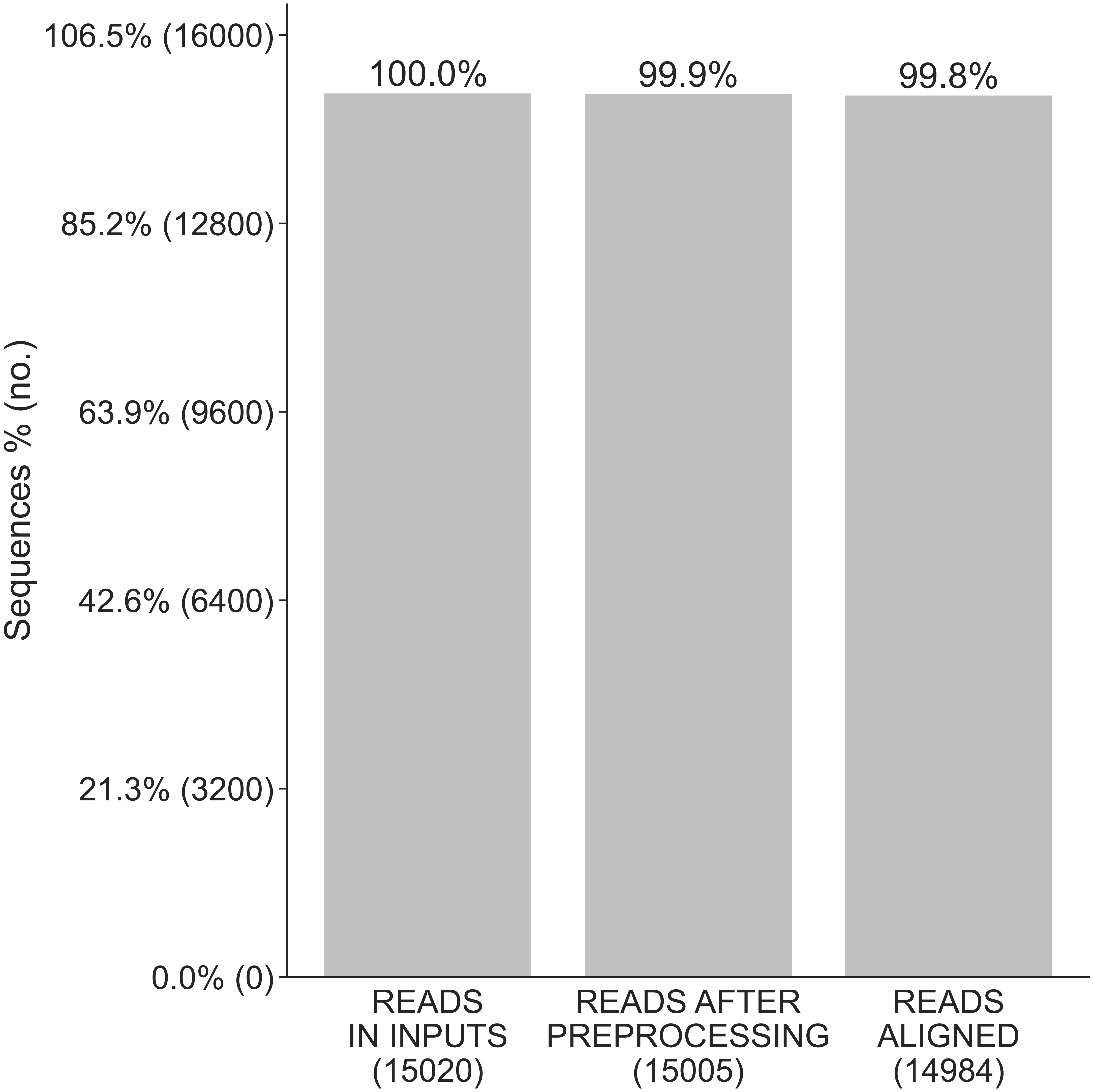
Sequences % (no.)
106.5% (16000)
85.2% (12800)
63.9% (9600)
42.6% (6400)
21.3% (3200)
0.0% (0)
100.0%
99.9%
99.8%
READS IN INPUTS (15020)
READS AFTER PREPROCESSING (15005)
READS ALIGNED (14984)
